# Optimal Pediatric Outpatient Antibiotic Prescribing

**DOI:** 10.1001/jamanetworkopen.2024.37409

**Published:** 2024-10-03

**Authors:** Brittany J. Lehrer, Glodi Mutamba, Katie A. Thure, Christopher D. Evans, Adam L. Hersh, Ritu Banerjee, Sophie E. Katz

**Affiliations:** 1 Division of Infectious Diseases, Department of Pediatrics, Vanderbilt University Medical Center, Nashville, Tennessee; 2 Healthcare-Associated Infections and Antimicrobial Resistance Program of the Communicable and Environmental Diseases and Emergency Preparedness Division, Tennessee Department of Health, Nashville; 3 Division of Infectious Diseases, Department of Pediatrics, University of Utah, Salt Lake City

## Abstract

**Importance:**

In the US, 50% of all pediatric outpatient antibiotics prescribed are unnecessary or inappropriate. Less is known about the appropriateness of pediatric outpatient antibiotic prescribing.

**Objective:**

To identify the overall percentage of outpatient antibiotic prescriptions that are optimal according to guideline recommendations for first-line antibiotic choice and duration.

**Design, Setting, and Participants:**

This cross-sectional study obtained data on any clinical encounter for a patient younger than 20 years with at least 1 outpatient oral antibiotic, intramuscular ceftriaxone, or penicillin prescription filled in the state of Tennessee from January 1 to December 31, 2022, from IQVIA’s Longitudinal Prescription Claims and Medical Claims databases. Each clinical encounter was assigned a single diagnosis corresponding to the lowest applicable tier in a 3-tier antibiotic tier system. Antibiotics prescribed for tier 1 (nearly always required) or tier 2 (sometimes required) diagnoses were compared with published national guidelines. Antibiotics prescribed for tier 3 (rarely ever required) diagnoses were considered to be suboptimal for both choice and duration.

**Main Outcomes and Measures:**

Primary outcome was the percentage of optimal antibiotic prescriptions consistent with guideline recommendations for first-line antibiotic choice and duration. Secondary outcomes were the associations of optimal prescribing by diagnosis, suboptimal antibiotic choice, and patient- and clinician-level factors (ie, age and Social Vulnerability Index) with optimal antibiotic choice, which were measured by odds ratios (ORs) and 95% CIs calculated using a multivariable logistic regression model.

**Results:**

A total of 506 633 antibiotics were prescribed in 488 818 clinical encounters (for 247 843 females [50.7%]; mean [SD] age, 8.36 [5.5] years). Of these antibiotics, 21 055 (4.2%) were for tier 1 diagnoses, 288 044 (56.9%) for tier 2 diagnoses, and 197 660 (39.0%) for tier 3 diagnoses. Additionally, 194 906 antibiotics (38.5%) were optimal for antibiotic choice, 259 786 (51.3%) for duration, and 159 050 (31.4%) for both choice and duration. Acute otitis media (AOM) and pharyngitis were the most common indications, with 85 635 of 127 312 (67.3%) clinical encounters for AOM and 42 969 of 76 865 (55.9%) clinical encounters for pharyngitis being optimal for antibiotic choice. Only 257 of 4472 (5.7%) antibiotics prescribed for community-acquired pneumonia had a 5-day duration. Optimal antibiotic choice was more likely in patients who were younger (OR, 0.98; 95% CI, 0.98-0.98) and were less socially vulnerable (OR, 0.84; 95% CI, 0.82-0.86).

**Conclusions and Relevance:**

This cross-sectional study found that less than one-third of antibiotics prescribed to pediatric outpatients in Tennessee were optimal for choice and duration. Four stewardship interventions may be targeted: (1) reduce the number of prescriptions for tier 3 diagnoses, (2) increase optimal prescribing for AOM and pharyngitis, (3) provide clinician education on shorter antibiotic treatment courses for community-acquired pneumonia, and (4) promote optimal antibiotic prescribing in resource-limited settings.

## Key Points

**Question:**

In Tennessee, what percentage of pediatric outpatient antibiotic prescriptions are consistent with first-line guideline recommendations, and what are the highest-yield targets for antibiotic stewardship?

**Findings:**

In this cross-sectional study of 506 633 antibiotics prescribed in clinical encounters in 2022 in Tennessee, only 31.4% of these antibiotics were optimal for both antibiotic choice and duration of therapy. Optimal antibiotic choice was more likely in patients who were younger and were less socially vulnerable.

**Meaning:**

Findings of this study suggest that few pediatric outpatient antibiotic prescriptions were optimal for both choice and duration; future outpatient stewardship efforts include reducing or increasing antibiotic prescribing for certain diagnoses.

## Teaser

This cross-sectional study examines the factors associated with optimal antibiotic choice, duration, and both choice and duration among outpatients younger than 20 years in Tennessee.

## Introduction

Antibiotics are among the most commonly prescribed medications in US individuals younger than 18 years, with a national average of 598 prescriptions per 1000 persons in 2022.^1^ Clinicians in the Southeast prescribe the highest volume of antibiotics per capita nationwide, and the state of Tennessee consistently ranks among the top 10 highest prescribing states.^2,3,4^ In 2016, the antibiotic prescribing rate for pediatric outpatients in Tennessee was 50% higher than the national average.^5^ Downstream outcomes of antibiotic overuse include antimicrobial resistance, adverse medication effects, and chronic conditions secondary to microbiome alterations, such as inflammatory bowel disease and asthma.^2,6,7,8^ Additionally, it is estimated that 80% of antibiotic consumption occurs in the outpatient setting.^9,10,11^ Thus, outpatient antimicrobial stewardship is of particular importance, and identifying factors in suboptimal antibiotic prescribing will allow health departments and stewardship programs in high-prescribing states to target, design, and implement future interventions.^12^

Despite national efforts to reduce the quantity and improve the quality of outpatient antibiotic prescribing, the estimated percentage of visits with prescribed antibiotics remains high (15%-34% of clinical encounters).^13,14,15^ The US Centers for Disease Control and Prevention (CDC) and other institutions estimate that 50% or more of prescribed antibiotics are either unnecessary or inappropriate.^16^ Typically, such studies estimate antibiotic appropriateness using a previously developed antibiotic tier system^11^ or evaluate appropriateness for specific disease processes, such as acute respiratory infections.^10,11,13,17,18,19^ However, these studies often define appropriateness based on clinician-level or encounter-level prescribing rates^10,11,19,20^ or the presence of any antibiotic prescription.^17,18^ Some studies evaluate optimal antibiotic choice,^13,21,22^ but few studies consider both optimal antibiotic choice and duration.^23,24^ Evaluations are lacking of optimal antibiotic prescribing at the clinical encounter level across an entire state. The primary objective of this study was to identify the overall percentage of outpatient antibiotic prescriptions that are optimal according to guideline recommendations for first-line antibiotic choice and duration.

## Methods

### Study Design and Data Sources

We performed a retrospective cross-sectional study to evaluate optimal pediatric antibiotic prescriptions in Tennessee. Patient diagnosis and antibiotic prescription data were collected from the IQVIA Medical Claims and Longitudinal Prescription Claims databases.^25^ The IQVIA Medical Claims database contains unadjudicated outpatient medical claims data inclusive of all *International Statistical Classification of Diseases, Tenth Revision, Clinical Modification* (*ICD-10-CM*) diagnosis codes assigned by clinicians to each clinical encounter. These data are collected from health insurers, medical record vendors, and coding centers. The IQVIA Longitudinal Prescription Claims database captures over 90% of retail pharmacy prescriptions and includes the name, dose, frequency, and days’ supply of the medications.^25^ The 2 databases were merged, with the parent database being IQVIA Longitudinal Prescription Claims, using a deidentified, unique patient identifier with a pharmacy fill date within 7 days of the office visit. The Vanderbilt University Institutional Review Board deemed this study exempt from review and waived the informed consent requirement because deidentified data were used. We followed the Strengthening the Reporting of Observational Studies in Epidemiology (STROBE) reporting guideline.^26^

### Inclusion and Exclusion Criteria

We included any clinical encounter for a patient younger than 20 years with at least 1 oral antibiotic, intramuscular ceftriaxone, or intramuscular penicillin prescription filled at a retail pharmacy in Tennessee from January 1 to December 31, 2022. We excluded visit diagnoses that were too broad (eg, encounter for health counseling related to travel), had no widely accepted antibiotic treatment guidelines (eg, mediastinitis), were related to pregnancy (all *ICD-10-CM* diagnosis codes beginning with O), were associated with an immunocompromising condition (eg, Crohn disease of small intestines with abscess), or had fewer than 50 clinical encounters in the dataset (eTable 1 in Supplement 1). We included antibiotic courses that were fully administered in the outpatient setting as well as courses that were started during inpatient stay but were completed in the outpatient setting.

### Visit Diagnosis

Prior to data analysis, we adapted an antibiotic tier system originally created by Fleming-Dutra et al^11^ from the *ICD-9-CM* (*International Classification of Diseases, Ninth Revision, Clinical Modification*) to the *ICD-10-CM* diagnosis codes (eTable 1 in Supplement 1) and added first-line antibiotic choice and duration recommendations from published guidelines. In this system, tier 1 diagnoses were considered to nearly always require antibiotics, tier 2 diagnoses sometimes require antibiotics, and tier 3 diagnoses rarely ever require antibiotics.^11^ We assessed all clinician-assigned *ICD-10-CM* diagnosis codes for a clinical encounter and categorized them into tiers. We then assigned a single overall visit diagnosis for each encounter using the clinician-assigned *ICD-10-CM* diagnosis code with the lowest suitable tier. Examples of visit diagnoses that were evaluated include acne, acute and chronic sinusitis, acute otitis media (AOM), community-acquired pneumonia (CAP), conjunctivitis, gastroenteritis, *Helicobacter pylori* infections, lymphadenitis, otitis externa, pharyngitis, sexually transmitted infections, and urinary tract infections (UTIs) (eTable 1 in Supplement 1).

When there was a specific tier 2 diagnosis (eg, acute pharyngitis) listed with a broad tier 1 diagnosis (eg, bacterial infection), the overall visit diagnosis was then attributed to the specific tier 2 diagnosis. If there were multiple diagnoses from the same tier, priority was given to the diagnosis with the most options for optimal antibiotics listed in the guidelines and/or the longest optimal duration of therapy (eFigure 1 in Supplement 1). Antibiotics prescribed for clinical encounters in tiers 1 and 2 were then compared with those recommended by national guidelines to ascertain whether antibiotic choice and duration were optimal. Antibiotics prescribed for visits in tier 3 were deemed unnecessary and were always considered suboptimal for both antibiotic choice and duration (eFigure 2 in Supplement 1).

### Optimal Antibiotic Prescriptions

An antibiotic prescription was deemed optimal if it was consistent with guideline recommendations for first-line antibiotic choice, duration, and both choice and duration for the specific diagnosis. Diagnoses, associated *ICD-10-CM* diagnosis codes, and defined optimal antibiotic choice and duration of therapy were based on the eReferences in Supplement 1. When available, national guidelines from the American Academy of Pediatrics, Infectious Diseases Society of America, and CDC were used to establish optimal antibiotic therapy. Preference was given to pediatric national guidelines. If national guidelines were not available, widely cited articles (>20 citations on Web of Science) were used. The range of optimal antibiotic duration for all diagnoses was deemed optimal if it was less than or equal to the maximum duration outlined in the guidelines (optimal choice and optimal duration columns in eTable 1 in Supplement 1) to account for antibiotic courses that may have been started in the inpatient setting but were finished in the outpatient setting. Therefore, all prescriptions with a suboptimal duration in this analysis were antibiotic courses that were longer than recommended. For example, if amoxicillin-clavulanate were prescribed for 7 days for streptococcal pharyngitis, that antibiotic choice would be deemed suboptimal, duration of therapy would be optimal (based on our definition), and overall choice and duration would be suboptimal. If multiple antibiotics were written for a single clinical encounter, each antibiotic was considered separately.

When results of more recent large randomized clinical trials (RCTs) have led to recommendations for shorter duration of therapy than in existing guidelines (eg, 5 days of therapy for CAP^27,28,29^), we performed a secondary analysis using the newer RCT-based recommendations to evaluate the potential implications for antibiotic prescribing practices. Shorter courses of therapy for pediatric CAP were supported by high-quality evidence and were the focus of the secondary analysis, but we also gauged the use of shorter antibiotic duration for other conditions with some supporting evidence, including uncomplicated UTIs (5 days),^30^ skin and soft-tissue infections (SSTIs; 7 days),^31^ lymphadenitis (7 days),^31^ acute sinusitis (7 days),^31^ AOM (age <2 years: 10 days; age ≥2 years: 7 days),^31^ complicated UTIs (10 days),^31^ and intra-abdominal infections (7 days).^31^ The shorter courses of therapy supported by these studies are hereafter referred to as *contemporary duration*. All azithromycin prescriptions were excluded from this secondary analysis of contemporary duration because a 5-day duration is standard for azithromycin.

### Statistical Analysis

Descriptive statistics were used to characterize the primary outcome of overall optimal prescribing for antibiotic choice, duration, and both choice and duration as well as the secondary analyses of optimal antibiotic choice by indication and the antibiotics prescribed when antibiotic choice was suboptimal. To evaluate patient-level and clinician-level factors associated with optimal antibiotic choice prescribed for tier 1 or 2 diagnoses, we used a multivariable logistic regression model.

The model focused on antibiotic choice because our broad definition of optimal duration made it uncertain whether the antibiotic treatment course was administered entirely in the outpatient setting. Independent variables included in the model were patient age (continuous), sex (dichotomous), clinician specialty (pediatrics vs other specialties), antibiotic indication (nominal), and the CDC Social Vulnerability Index (SVI)^32^ of the pharmacy zip code (continuous) using the 2020 US Census Bureau information. The SVI is a composite score of 16 US Census variables, including but not limited to poverty, access to transportation, and crowded housing, that indicates the potential degree of adversity a community experiences due to external stressors.^32^ For the logistic regression analysis, optimal prescriptions were defined at the clinical encounter level; therefore, if multiple antibiotics were prescribed during the same encounter and at least 1 was deemed to be optimal, treatment for the encounter was considered to be optimal.

Two-sided *P* < .05 indicated statistical significance. Data were analyzed using SAS, version 9.4 (SAS Institute Inc).

## Results

### Clinical Encounters and Patient Demographics

**Figure 1.**
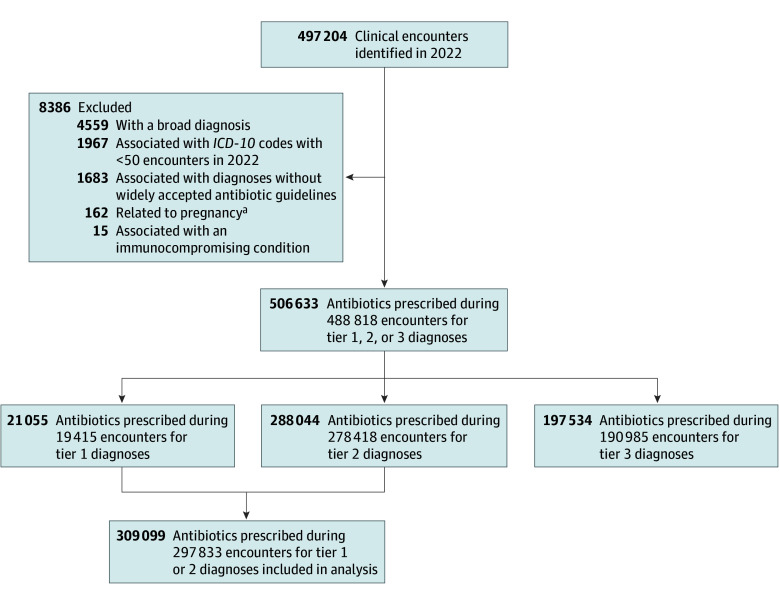
Diagram of Analyzed Antibiotic Prescriptions and Encounters *ICD-10* indicates *International Statistical Classification of Diseases, Tenth Revision.* ^a^*ICD-10* diagnosis codes beginning with O.

**Figure 2.**
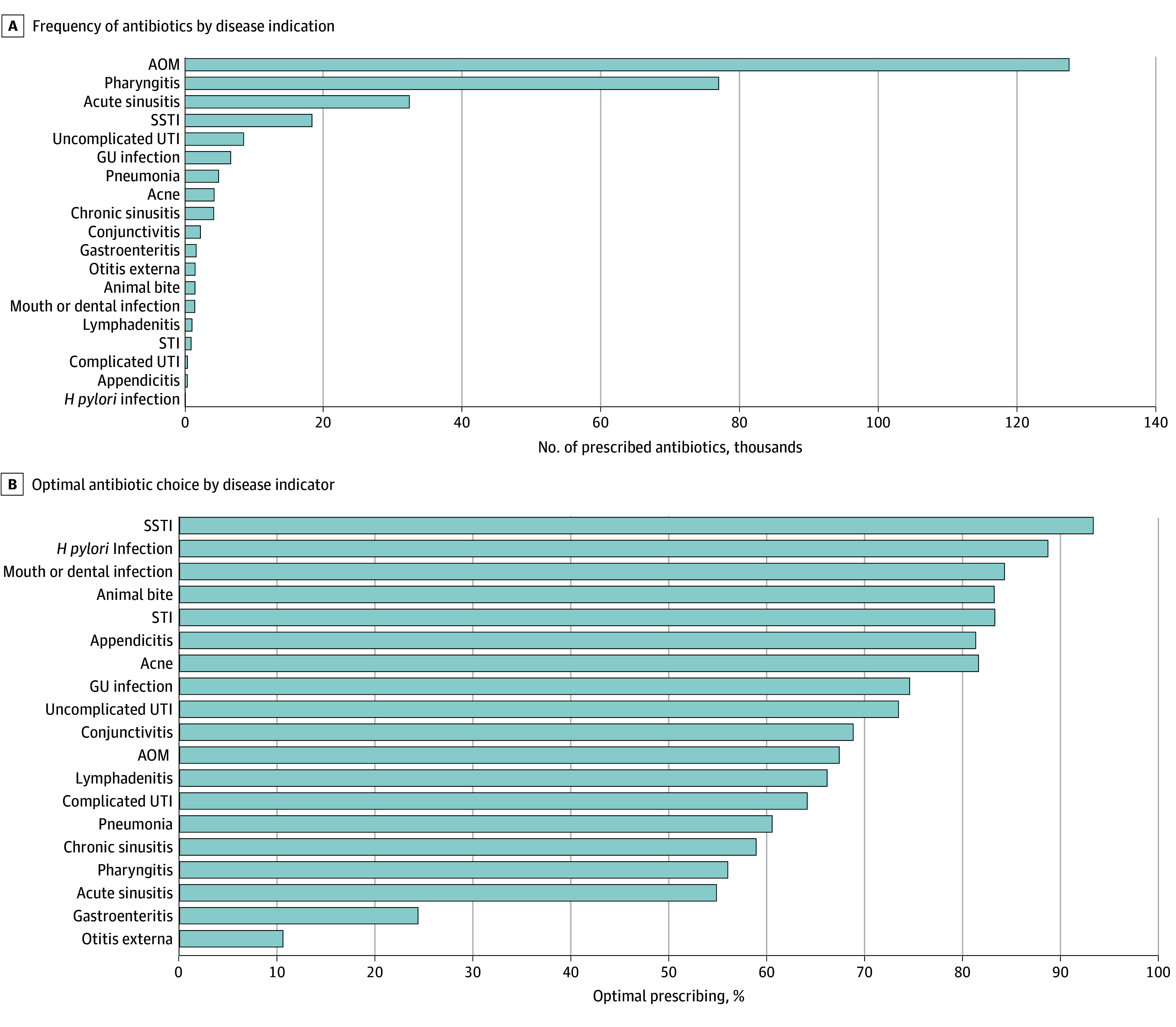
Frequency of Antibiotics and Percentage of Optimal Antibiotic Choice by Indication AOM indicates acute otitis media; GU, genitourinary; *H pylori, Helicobacter pylori*; SSTI, skin and soft-tissue infection; STI, sexually transmitted infection; and UTI, urinary tract infection.

In 2022, 506 633 antibiotics were prescribed in 488 818 clinical encounters for 247 843 females (50.7%) and 240 975 males (49.3%), with a mean (SD) age of 8.36 (5.5) years. Of these antibiotics, 21 055 (4.2%) were for tier 1 diagnoses, 288 044 (56.9%) for tier 2 diagnoses, and 197 534 (39.0%) for tier 3 diagnoses (Figure 1). A total of 471 486 clinical encounters (96.5%) were associated with only 1 antibiotic prescription, and 1.0% of prescriptions (3073 antibiotics prescribed for tier 1 or 2 diagnoses) were prescribed for a duration of fewer than 5 days. Nurse practitioners accounted for the highest proportion of prescribers (196 324 encounters [40.2%]) followed by general pediatricians (168 796 encounters [34.5%]) (Table 1). The 3 most common indications for antibiotic prescriptions were AOM (127 312 encounters [26.0%]), pharyngitis (76 865 encounters [15.7%]), and acute sinusitis (32 307 encounters [6.6%]) (Table 1, Figure 2A). The 3 most frequently prescribed antibiotics were amoxicillin (37.1%), cefdinir (18.3%), and amoxicillin-clavulanate (13.9%).

**Table 1.** Demographic and Clinical Encounter Characteristics

Characteristic	Encounters, No. (%)		
	Total (N = 488 818)	Tier of diagnoses	
		1 and 2 (n = 297 833)	3 (n = 190 985)
Age group, y			
0-2	73 435 (15.0)	46 176 (15.5)	27 259 (14.3)
3-9	234 835 (48.0)	149 481 (50.2)	85 354 (44.7)
10-19	180 548 (36.9)	102 176 (34.3)	78 372 (41.0)
Patient sex			
Female	247 843 (50.7)	153 024 (51.4)	94 819 (49.6)
Male	240 975 (49.3)	144 809 (48.6)	96 166 (50.4)
SVI			
0-0.2599: least vulnerable	145 031 (29.7)	91 358 (30.7)	53 673 (28.1)
0.2600-0.5099: slightly vulnerable	116 782 (23.9)	69 678 (23.7)	47 104 (24.7)
0.5100-0.7599: somewhat vulnerable	107 685 (22.0)	64 702 (21.7)	42 983 (22.5)
0.7600-1.000: most vulnerable	119 320 (24.4)	72 095 (24.2)	47 225 (24.7)
Clinician specialty			
Nurse practitioner, all specialties	196 324 (40.2)	122 495 (41.1)	73 829 (38.7)
General pediatrician	168 796 (34.5)	111 787 (37.5)	57 009 (29.8)
Physician assistant, all specialties	46 698 (9.6)	29 516 (9.9)	17 182 (9.0)
Pediatric subspecialist	17 965 (3.7)	6889 (2.3)	11 076 (5.8)
Family medicine	17 877 (3.7)	9789 (3.3)	8088 (4.2)
Emergency medicine	9736 (2.0)	5594 (1.9)	4142 (2.2)
Internal medicine	8743 (1.8)	4720 (1.6)	4023 (2.1)
General surgery	4284 (0.9)	648 (0.2)	3636 (1.9)
Dentist	3033 (0.6)	235 (0.1)	2798 (1.5)
Dermatology	2355 (0.5)	1528 (0.5)	827 (0.4)
Medicine subspecialist	2467 (0.5)	982 (0.3)	1485 (0.8)
Podiatrist	934 (0.2)	477 (0.2)	457 (0.2)
Obstetrics/gynecology	762 (0.2)	311 (0.1)	451 (0.2)
Urology	702 (0.1)	180 (0.1)	522 (0.3)
Trainees, all specialties	507 (0.1)	214 (0.1)	293 (0.2)
Optometry	314 (0.1)	141 (0)	173 (0.1)
Veterinarian	27 (0)	6 (0)	21 (0)
Sports medicine	4 (0)	2 (0)	2 (0)
Other	5986 (1.2)	1832 (0.6)	4154 (2.2)
Missing data	1304 (0.3)	487 (0.2)	817 (0.4)
No. of encounters with antibiotic prescriptions			
1 Prescription	471 486 (96.5)	286 847 (96.3)	184 639 (96.7)
>1 Prescription	17 332 (3.6)	10 710 (4.0)	6346 (3.3)
No. of encounters with *ICD-10-CM* codes assigned			
1 Code	332 343 (68.0)	253 790 (85.2)	78 553 (41.1)
>1 Code	156 475 (32.0)	44 043 (14.8)	112 432 (58.9)
Visit diagnoses (only tiers 1 and 2)			
AOM	127 312 (26.0)	127 312 (42.7)	NA
Pharyngitis	76 865 (15.7)	76 865 (25.8)	NA
Acute sinusitis	32 307 (6.6)	32 307 (10.8)	NA
SSTI	18 291 (3.7)	18 291 (6.1)	NA
Uncomplicated UTI	8428 (1.7)	8428 (2.8)	NA
GU infection	6582 (1.3)	6582 (2.2)	NA
Pneumonia	5694 (1.2)	5694 (1.9)	NA
Acne	4950 (1.0)	4950 (1.7)	NA
Chronic sinusitis	4855 (1.0)	4855 (1.6)	NA
Conjunctivitis	2616 (0.5)	2616 (0.9)	NA
Gastroenteritis	1864 (0.4)	1864 (0.9)	NA
Mouth or dental infection	1655 (0.3)	1655 (0.6)	NA
Animal bite	1683 (0.3)	1683 (0.6)	NA
Otitis externa	1698 (0.3)	1698 (0.6)	NA
Lymphadenitis	1171 (0.2)	1171 (0.4)	NA
STI	1013 (0.2)	1013 (0.3)	NA
Complicated UTI	411 (0.1)	411 (0.1)	NA
Appendicitis	377 (0.1)	377 (0.1)	NA
*Helicobacter pylori* infection	61 (0)	61 (0)	NA

Abbreviations: AOM, acute otitis media; GU, genitourinary; *ICD-10-CM, International Statistical Classification of Diseases, Tenth Revision, Clinical Modification*; NA, not applicable; SSTI, skin and soft-tissue infection; STI, sexually transmitted infection; SVI, Social Vulnerability Index; UTI, urinary tract infection.

### Overall Optimal Antibiotic Prescribing and Antibiotic Choice by Indication

Among the 506 633 antibiotics prescribed, 194 906 (38.5%) were optimal for antibiotic choice, 259 786 (51.3%) were optimal for duration of therapy, and 159 050 (31.4%) were optimal for both antibiotic choice and duration. After exclusion of 197 534 (39.0%) antibiotics prescribed for tier 3 diagnoses only (Figure 1), the 309 099 antibiotics remaining were prescribed for tiers 1 and 2 diagnoses; among these medications, 159 050 (51.5%) were optimal for both antibiotic choice and duration. The most common tier 3 diagnosis for which an antibiotic was prescribed was an unspecified acute upper respiratory infection with over 20 000 prescriptions (eTable 2 in Supplement 1).

When evaluating antibiotic choice by indication, antibiotics prescribed for SSTIs were most frequently optimal (17 039 of 18 291 [93.2%]) encounters (Figure 2B). The diagnosis with the lowest percentage of optimal antibiotic choice was otitis externa (179 of 1698 encounters [10.5%]), followed by gastroenteritis (453 of 1864 encounters [24.3%]) and acute sinusitis (17 686 of 32 307 encounters [54.7%]) (Figure 2B). Cefdinir was the antibiotic most often prescribed as a suboptimal choice, with 48 523 prescriptions among the 3 most common diagnoses of AOM, pharyngitis, and sinusitis (eFigure 3 in Supplement 1). Acute otitis media received an optimal antibiotic choice in 85 635 of 127 312 (67.3%) clinical encounters, and pharyngitis received an optimal antibiotic choice in 42 969 of 76 865 (55.9%) clinical encounters.

### Optimal Antibiotic Duration by Indication and Based on Standard vs Contemporary Duration

When evaluating optimal antibiotic duration outlined in the standard guidelines, 259 786 of 506 633 clinical encounters (51.3%) were prescribed optimally. After excluding all prescriptions for tier 3 diagnoses, optimal antibiotic duration increased to 259 786 of 309 099 encounters (84.0%). The indication with the most suboptimal duration of therapy was for gastroenteritis (optimal duration of ≤14 days based on the associated *ICD-10-CM* diagnosis code), with just 167 of 1864 clinical encounters (9.0%) prescribed optimally.

**Figure 3.**
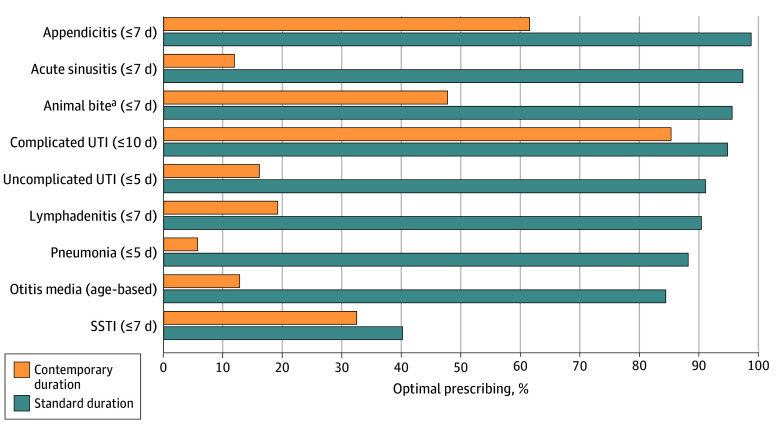
Percentage of Standard vs Contemporary Antibiotic Duration Optimal contemporary duration of antibiotic prescribing defined in parentheses. UTI indicates urinary tract infection. ^a^Extrapolated from expert opinion on shorter duration for skin and soft-tissue infection (SSTI).

After excluding azithromycin prescriptions and restricting the clinical encounters to indications of AOM, animal bites, appendicitis, complicated and uncomplicated UTI, lymphadenitis, pneumonia, SSTI, and sinusitis, 183 818 encounters were used to evaluate optimal antibiotic duration based on contemporary duration. Of these clinical encounters, 27 973 (15.2%) aligned with contemporary duration. Specifically, the number of pneumonia encounters for optimal duration of therapy decreased from 5025 of 5694 (88.3%) under standard duration (10 days) to 257 of 4472 (5.7%) under contemporary (5 days) duration (Figure 3).

### Multivariable Logistic Regression for Optimal Antibiotic Choice

The multivariable logistic regression model evaluating optimal antibiotic choice included 297 833 tiers 1 and 2 clinical encounters. Patient age (odds ratio [OR], 0.98; 95% CI, 0.98-0.98; *P* < .001), antibiotic indication (eg, genitourinary infection: OR, 1.63; 95% CI, 1.54-1.73; *P* < .001), and SVI (OR, 0.84; 95% CI, 0.82-0.86; *P* < .001) were found to be significant independent factors of optimal antibiotic prescribing. For every 1-year increase in age, the odds of being prescribed an optimal antibiotic decreased by 2.2%, and for each 0.1 increase in SVI, the odds of being prescribed an optimal antibiotic decreased by 1.5%. Clinician specialty (pediatrics vs other specialties) and patient sex were not significant factors of optimal antibiotic choice (Table 2).

**Table 2.** Multivariable Logistic Regression for Optimal Antibiotic Choice

Characteristic	2022 Encounters (n = 297 833)	
	OR (95% CI)	*P* value^a^
Demographics		
Age	0.98 (0.98-0.98)	<.001
Sex^b^	1.01 (0.99-1.02)	.34
Clinician specialty^c^	0.99 (0.98-1.01)	.29
SVI	0.84 (0.82-0.86)	<.001
Indication		
AOM	1 [Reference]	NA
GU infection	1.63 (1.54-1.73)	<.001
*Helicobacter pylori* infection	4.41 (2.00-9.70)	<.001
STI	3.24 (2.74-3.83)	<.001
Acne	2.75 (2.55-2.97)	<.001
Animal bite	2.67 (2.35-3.04)	<.001
Appendicitis	2.53 (1.95-3.27)	<.001
Chronic sinusitis	0.78 (0.74-0.83)	<.001
Complicated UTI	1.00 (0.81-1.22)	.04
Conjunctivitis	1.07 (0.99-1.17)	.01
Gastroenteritis	0.16 (0.15-0.18)	<.001
Lymphadenitis	1.03 (0.91-1.16)	<.001
Mouth or dental infection	3.00 (2.62-3.42)	<.001
Otitis externa	0.06 (0.06-0.08)	<.001
Pharyngitis	0.69 (0.67-0.70)	<.001
Pneumonia	0.76 (0.72-0.81)	<.001
Acute sinusitis	0.67 (0.65-0.69)	<.001
SSTI	7.43 (7.00-7.89)	<.001
Uncomplicated UTI	1.53 (1.46-1.61)	<.001

Abbreviations: AOM, acute otitis media; GU, genitourinary; NA, not applicable; OR, odds ratio; SSTI, skin and soft-tissue infection; STI, sexually transmitted infection; SVI, Social Vulnerability Index; UTI, urinary tract infection.

^a^ *P* < .05 was statistically significant.

^b^ Female (reference) vs male.

^c^ Pediatrician (reference) vs other specialties.

## Discussion

In Tennessee, despite the availability of several treatment guidelines for years, less than one-third of the antibiotic prescriptions (31.4%) were optimal in both choice and duration. Optimal prescribing percentages decreased across all diagnoses when evaluated against shorter treatment duration for several infections.^27,28,29,30,31,33^

This cross-sectional study identified several factors in suboptimal pediatric outpatient antibiotic prescribing. First, the large number of antibiotics prescribed for tier 3 diagnoses only (197 534 [39.0%]) was almost entirely unnecessary. This finding has also been consistent with previous data from the Tennessee Department of Health.^34^ Drastically reducing the number of prescriptions for tier 3 diagnoses would increase optimal prescribing to approximately 50%. Second, antibiotics prescribed for AOM and pharyngitis made up over two-thirds of the tiers 1 and 2 prescriptions but were the optimal choice only 67.3% and 55.9% of the time, respectively. While we do not expect 100% optimal prescribing rates (approximately 10% of patients have a penicillin allergy,^35,36^ and some pediatric patients with AOM experience treatment failure^37^), there is room for improvement in antibiotic selection for these common diagnoses. If optimal antibiotic choice for these 2 diagnoses increased to approximately 80%, that would equate to over 163 000 optimal prescriptions annually for 1 state. Third, as supporting evidence develops and shorter therapy duration become accepted and recommended by guidelines, outreach to clinicians may be needed to encourage uptake. At the time of the present study, several RCTs had reported on the safety and effectiveness of a 5-day duration of therapy for uncomplicated CAP^27,28,29^; however, we found that only 5.7% of prescriptions were prescribed for 5 days. Data from these RCTs had not yet been incorporated into the existing pediatric CAP treatment guidelines during the study period, likely affecting the low uptake of shorter treatment duration and emphasizing the importance of education and outreach to clinicians.

Results of the multivariable logistic regression model demonstrated that younger age, diagnosis, and areas with low SVI were factors in whether the patient would be prescribed an optimal antibiotic (choice). We hypothesized that clinicians may be more likely to prescribe optimal antibiotics for toddlers (vs teens) because they have a lower incidence of antibiotic allergy, are more often antibiotic naive, or have different health care–seeking patterns.^5,38^ Additionally, pharmacy location in an area with low SVI (surrogate of patient residence) was a significant factor in optimal antibiotic prescribing. More studies are needed to explore this relationship, as some studies^39,40,41^ have shown that racial and ethnic minoritized populations receive fewer but more appropriate antibiotics, whereas other studies^17,42,43^ have found that racial and ethnic minority groups and rural populations are more likely to receive suboptimal antibiotics. The reasons for this discrepancy are likely multifactorial and, as Cichon et al^42^ stated, may include lack of access to quality diagnostic resources, lack of prescriber training, overburdened clinics, or pharmacy deserts.

### Strengths and Limitations

This study has several strengths. First, we evaluated a large sample of antibiotics prescribed for several indications across an entire state. These prescriptions were from many clinic settings and included written, electronic, and telephone prescriptions. Second, this study complements previous work in this domain by providing granularity regarding optimal antibiotic prescribing for tier 1 and tier 2 diagnoses that, to our knowledge, has not been available at the state level. Third, we used SVI as a marker of health equity to evaluate the disparities in optimal antibiotic prescribing. We created a resource (eTable 1 in Supplement 1) that includes *ICD-10-CM* diagnosis codes with associated guideline recommendations for first-line antibiotic choice and duration. Fourth, this work outlines specific, high-yield stewardship targets that health departments, clinics, hospitals, health systems, or payers could focus on to improve optimal antibiotic prescribing.

This study also has several limitations. First, by using 2 IQVIA databases that required matching and by analyzing only antibiotic prescriptions with corresponding diagnoses, we risked overestimating optimal prescribing. Second, the assigned visit diagnosis was based on clinician-selected *ICD-10-CM* diagnosis codes.^44^ We were not able to clinically adjudicate any diagnoses or distinguish prescriptions for prophylaxis vs those for treatment; therefore, we risked misclassifying indications. Third, the assigned indications did not account for disease severity and, when there were multiple *ICD-10-CM* diagnosis codes, the visit diagnosis may not reflect the clinician’s intended indication for antibiotics. Fourth, if multiple antibiotics were prescribed during a single visit, each antibiotic was considered separately for the visit diagnosis and therefore may overestimate or underestimate the amount of optimal antibiotics prescribed. Fifth, we were not able to track antibiotics prescribed while patients were hospitalized and therefore risked misclassifying the duration of therapy for patients who initially received antibiotics as an inpatient but finished their antibiotic course as an outpatient. This risk is likely trivial given that only 1.0% of prescriptions were prescribed for fewer than 5 days. Sixth, we were not able to account for antibiotic allergies, which is likely a common reason for suboptimal prescribing. Seventh, in this study, optimal acne duration was defined as 365 days or fewer; however, the American Academy of Dermatology recommends 4 months or fewer. This definition was an oversight in the analysis and may overestimate the amount of optimal antibiotics prescribed. Eighth, there were national antibiotic shortages for many antibiotic suspensions in the fourth quarter of 2022, which may have prompted clinicians to use alternative therapies.^45^ Ninth, while the findings were directly applicable to Tennessee and likely generalizable to other high-prescribing states in the Southeast US, they may not be generalizable to other areas of the country.

## Conclusions

Less than one-third of antibiotics prescribed to pediatric outpatients in Tennessee were optimal for choice and duration. The findings of this study highlight a substantial opportunity to achieve optimal outpatient antibiotic prescriptions for these patients. Future stewardship interventions in Tennessee and other high-prescribing states in the Southeast US include (1) reducing antibiotic prescriptions for tier 3 diagnoses, (2) increasing antibiotic prescribing for AOM and pharyngitis, (3) providing clinician education on shorter antibiotic treatment courses for CAP, and (4) promoting optimal antibiotic prescribing in resource-limited settings.

## Notes

Supplement 1. **eTable 1.** Optimal Antibiotic Choice and Durations of Therapy by Diagnosis **eFigure 1.** Hierarchy of ICD-10 Codes When Assigning Single Visit Diagnosis to Each Encounter **eFigure 2.** Algorithm for Determining Optimal Antibiotics for Each Encounter **eFigure 3.** Most Common Diagnoses and the Suboptimal Antibiotics That Were Prescribed Instead of Optimal Therapies (Based on Antibiotic Choice) **eTable 2.** Most Common Tier 3 Diagnoses With At Least 1 Antibiotic Prescribed
eReferences

Supplement 2.
Data Sharing Statement
