# Supplementary material for: Optimal Pediatric Outpatient Antibiotic Prescribing
Source: JAMA Netw Open. 2024 Oct 3;7(10):e2437409. doi: 10.1001/jamanetworkopen.2024.37409 (PMC11450517; doi:10.1001/jamanetworkopen.2024.37409)
Supplement: Supplement 1. — eTable 1. Optimal Antibiotic Choice and Durations of Therapy by Diagnosis eFigure 1. Hierarchy of ICD-10 Codes When Assigning Single Visit Diagnosis to Each Encounter eFigure 2. Algorithm for Determining Optimal Antibiotics for Each Encounter eFigure 3. Most Common Diagnoses and the Suboptimal Antibiotics That Were Prescribed Instead of Optimal Therapies (Based on Antibiotic Choice) eTable 2. Most Common Tier 3 Diagnoses With At Least 1 Antibiotic Prescribed eReferences [file jamanetwopen-e2437409-s001.pdf]

Supplemental Online Content

Lehrer BJ, Mutamba G, Thure KA, et al. Optimal pediatric outpatient antibiotic prescribing. *JAMA Netw Open*. 2024;7(10):e2437409.
doi:10.1001/jamanetworkopen.2024.37409

eTable 1. Optimal Antibiotic Choice and Durations of Therapy by Diagnosis

eFigure 1. Hierarchy of ICD-10 Codes When Assigning Single Visit Diagnosis to Each Encounter

eFigure 2. Algorithm for Determining Optimal Antibiotics for Each Encounter

eFigure 3. Most Common Diagnoses and the Suboptimal Antibiotics That Were Prescribed Instead of Optimal Therapies (Based on Antibiotic Choice)

eTable 2. Most Common Tier 3 Diagnoses With At Least 1 Antibiotic Prescribed

eReferences

This supplemental material has been provided by the authors to give readers additional information about their work.

eTable 1. Optimal Antibiotic Choice and Durations of Therapy by Diagnosis

Indication	ICD-10 codes	Standard Guidelines: Optimal Choice[a]	Standard Guidelines: Optimal Duration[b] (days)	Standard Guidelines: Reference	Contemporary Guidelines[c]: Optimal Duration[b] (days)	Contemporary Guidelines[c]: Reference
**Tier 1 Diagnoses – Antibiotics are almost always indicated**						
Complicated urinary tract infection	A02.25, N10-N12, N13.6, N15.1	Ceftriaxone<br>Cefixime<br>Cephalexin<br>Ciprofloxacin<br>Levofloxacin<br>TMP-SMX	7-14<br>7-14<br>7-14<br>7-14<br>7-14<br>7-14	Roberts 2011[1], Gupta 2011[2]	10<br>10<br>10<br>10<br>10<br>10	AAP 2021 Red Book[3]
Helicobacter pylori infection	B96.81	Amoxicillin<br>Clarithromycin<br>Metronidazole	14<br>14<br>14	Jones 2017 NASPGHAN[4]	N/A	
Mouth/Dental infection	*(Cellulitis/abscess)*<br>J36, J39.0-J39.1, K04-K04.1, K04.4-K04.7, K11, K11.3-K11.4, K12.2	Amoxicillin<br>Amox-clav<br>Clindamycin<br>Metronidazole<br>Penicillin V	7-10<br>7-10<br>7-10<br>7-10<br>7-10	Nguyen 2008 AAFP[5]<br>Cionca 2009[6]	N/A	
	(Other general mouth/dental infections)<br>K04.90-K05, K05.2-K05.229, K05.4, K11.2-K11.21, K12.1, K12.3-K12.30	Amoxicillin<br>Clindamycin<br>Penicillin V	7-10<br>7-10<br>7-10	Nguyen 2008 AAFP[5]<br>Cionca 2009[6]	N/A	
Community Acquired Pneumonia	*(Acute pneumonia)*<br>J13, J15, J15.4, J15.8-J16, J16.8-J18.0, J18.2-J18.9	Amoxicillin<br>Azithromycin<br>Penicillin V	10<br>5<br>10	Bradley 2011 IDSA[7]	5<br>*Excluded*<br>5	Bielicki 2021[8], Pernica 2021[9], Williams 2022[10]
	(Pneumonia due to Mycoplasma spp.)<br>J15.7	Azithromycin	5	Bradley 2011 IDSA[7]	N/A	
	(Lobar pneumonia)<br>J18.1	Amoxicillin<br>Penicillin V	10<br>10	Bradley 2011 IDSA[7]	5<br>5	Bielicki 2021[8], Pernica 2021[9] Williams 2022[10]
Sexually transmitted infection (STI)	*(General)*<br>A50-A54.02, A54.09-A54.23, A54.29-A54.89, A55-A56.1, A56.19-A59.9, A63, A63.8-A64, A74.0-A74.8, A74.89-A74.9, Z11.3, Z20.2	Ceftriaxone<br>Doxycycline<br>Metronidazole<br>Penicillin G<br>Amoxicillin<br>Amox-clav	1-7<br>7<br>1-7<br>1-3<br>7-10<br>7-10	Workowski 2021 CDC[11]	N/A	

© 2024 Lehrer BJ et al. *JAMA Network Open*

Indication	ICD-10 codes	Standard Guidelines Optimal Choice[a]	Standard Guidelines Optimal Duration[b] (days)	Standard Guidelines Reference	Contemporary Guidelines[c] Optimal Duration[b] (days)	Contemporary Guidelines[c] Reference
		Cefixime<br>Cefpodoxime<br>Cephalexin<br>Nitrofurantoin<br>TMP-SMX	7-10<br>7-10<br>7-10<br>7-10<br>7-10			
	(Pelvic Inflammatory Disease)<br>A54.03, A54.24, A54.9, A56.11, A74.81	Ceftriaxone<br>Doxycycline<br>Metronidazole<br>Penicillin G<br>Amoxicillin<br>Amox-clav<br>Cefixime<br>Cefpodoxime<br>Cephalexin<br>Nitrofurantoin<br>TMP-SMX	1-14<br>14<br>14<br>1-3<br>7-10<br>7-10<br>7-10<br>7-10<br>7-10<br>7-10<br>7-10	Workowski 2021 CDC[11]	N/A	
Uncomplicated urinary tract infection (UTI)	N39.0	Amoxicillin<br>Amox-clav<br>Cefixime<br>Cefpodoxime<br>Ceftriaxone<br>Cephalexin<br>Nitrofurantoin<br>TMP-SMX	7-10<br>7-10<br>7-10<br>7-10<br>7-10<br>7-10<br>7-10<br>7-10	Roberts 2011[1]<br>Gupta 201[2]<br>Pediatric Outpatient Treatment Table 2023 CDC[12]	5<br>5<br>5<br>5<br>5<br>5<br>5<br>5	Zaoutis 2023[13]<br>AAP 2021 Red Book[3]
Excluded Tier 1 Diagnoses	A01-A02, A02.1-A02.24, A02.29-A02.9, A06, A15-A28.0, A28.2-A37.91, A39-A44.9, A48-A49.9, A63, A63.8, A65-A74, B47.1, B50-B54, B58-B60.09, B95-B96.2, B96.29-B96.8, B96.82-B96.89, D57.01, D57.211, D57.411, D57.431, D57.451, D57.811, E08.52, E09.52, E10.52, E11.52, E13.52, E32.1, G00.0-G03.1, G03.8-G04, G04.2-G04.30, G06-G08, G37.4, H05.01-H5.1, H05.12- H05.129, H10.22-H10.229, H44-H44.1, H44.19, H57.01, H70-H70.8, H70.819-H70.93, H75-H75.03, I00-I09.9, I38-I39, I70.26-I70.269, I70.36-I70.369, I70.46-I70.469, I70.56-I70.569, I70.66-I70.669, I70.76-I70.769, I73.01, I76, I80-I80.9, I96, J05, J05.1-J05.11, J14, J15.0-J15.3, J15.5-J15.69, J16.0, J18.2, J47-J47.1, J67.2, J84.116, J85-J86.9, J95.851, J98.51, K22.3, K25.1-K25.2, K25.5-K25.6, K26.1-K26.2, K26.5-K26.6, K27.1-K27.2, K27.5-K27.6, K28.1-K28.2, K28.5-K28.6, K40.1-K40.11, K40.4-K40.41, K41.1-K41.11, K41.4-K41.41, K42.1, K43.1, K43.4, K43.7, K44.1, K45.1, K46.1, K50.014, K50.114, K50.814, K50.914, K51.014, K51.214, K51.314, K51.414, K51.514, K51.814, K51.914, K55.3-K55.33, K57.0-K57.01, K57.12-K57.21, K57.32-K57.41, K57.8-K57.81, K59.3-K59.39, K60.3-K61.5, K63.0-K63.2, K63.8211-					

© 2024 Lehrer BJ et al. *JAMA Network Open*

Indication	ICD-10 codes	Standard Guidelines			Contemporary Guidelines[c]	
		Optimal Choice[a]	Optimal Duration[b] (days)	Reference	Optimal Duration[b] (days)	Reference
	K63.822, K65-K65.3, K65.8-K65.9, K68.1-K68.19, K75.0-K75.1, K80.3-K80.37, K82.2, K82.A-K82.A2, K83, K83.09, K83.2-K83.3, K90.81, K94.02, K94.12, K94.22, K94.32, K95.0-K95.02, K95.8-K95.81, L03.213, M00-M01.X9, M02.3-M02.39, M27.3, M27.51, M46.2-M46.39, M46.5-M46.59, M60-M60.09, M72.6, M86-M86.9, N41-N41.3, N43.1, N45.4, N49.3, N76.82, N98.0, N99.511, P23.1-P24.0, P36-P39.9, P77-P78.1, P78.8, R57.9, R65, R65.2-R65.21, R78.81, R83.4-R83.9, R88.0, all infections from T07-T88.9XXS, Z11, Z20-Z20.09, Z20.8-Z20.811, Z22-Z22.32, Z22.4, Z22.7					
**Tier 2 Diagnoses – Antibiotics are sometimes indicated**						
Acne	L70-L70.3, L70.5-L70.9, L73.0	Clindamycin<br>Doxycycline<br>Erythromycin<br>Minocycline	14-365<br>14-365<br>14-365<br>14-365	Johnson 2000 AAFP[14], Zaenglein 2016 AAD[15]	N/A	
Animal bite	*(Cat bites)*<br>A28.1, W55.01-W55.03XS	Amox-clav<br>Azithromycin	10<br>5	Stevens 2014 IDSA[16]	5-7<br>excluded	AAP 2021 Red Book[3] (extrapolated from cellulitis)
	(Bite of lip or cheek, bite from another person)<br>S00.57-S00.572S, S01.45-S01.459S, S01.55-S01.552S, W50.3-W50.3XXS	Amoxicillin<br>Amox-clav<br>Doxycycline	5-10<br>5-10<br>5-10	Stevens 2014 IDSA[16]	5-7<br>5-7<br>5-7	AAP 2021 Red Book[3] (extrapolated from cellulitis)
	(Animal bites, open bites of body part)<br>S00.07-S00.07XS, S00.27-S00.279S, S00.37-S00.37XS, S00.47-S00.479S, S00.87, S00.87XS, S00.97-S00.97XS, S01.05-S01.05XS, S01.15-S01.159S, S01.25-S01.25XS, S01.35-S01.359S, S01.85-S01.85XS, S01.95-S01.95XS, S10.17-S10.17XS, S10.87-S10.87XS, S10.97-S10.97XS, S11.015-S11.015S, S11.025-S11.025S, S11.035-S11.035S, S11.15-S11.15XS, S11.25-S11.25S, S11.85-S11.85XS, S11.95-S11.95S, S20.17-S20.179S, S20.37-S20.379S, S20.47-S20.479S, S20.97-S20.97XS, S21.05-S21.059S, S2115-S21.159S, S21.25-S21.259S, S21.35-S21.359S, S21.45-	Amox-clav	3-10	Stevens 2014 IDSA[16]	5-7	AAP 2021 Red Book[3] (extrapolated from cellulitis)

© 2024 Lehrer BJ et al. *JAMA Network Open*

Indication	ICD-10 codes	Standard Guidelines			Contemporary Guidelines[c]	
		Optimal Choice[a]	Optimal Duration[b] (days)	Reference	Optimal Duration[b] (days)	Reference
	S21.459S, S21.95-S21.95XS, S30.87-S30.877S, S31.05-S31.051S, S31.15-S31.159S, S31.25-S31.25XS, S31.35-S31.35XS, S31.45-S31.45XS, S31.55-S31.552S, S31.65-S31.659S, S31.805-S31.805S, S31.815-S31.815S, S31.825-S31.825S, S31.835-S31.835S, S40.27-S40.279S, S40.87-S40.879S, S41.05-S41.059S, S41.15-S41.159S, S50.37-S50.379S, S50.87-S50.879S, S51.05-S51.059S, S51.85-S51.859S, S60.47-S60.479S, S60.57-S60.579S, S60.87-S60.879S, S61.05-S61.059S, S61.15-S61.159S, S61.25-S61.259S, S61.35-S61.359S, S61.45-S61.459S, S61.55-S61.559S, S70.27-S70.279S, S70.37-S70.379S, S71.05-S71.059S, S71.15-S71.159S, S80.27-S80.279S, S80.87-S80.879S, S81.05-S81.059S, S81.85-S81.859S, S90.47-S90.476S, S90.57-S90.579S, S90.87-S90.879S, S91.05-S91.059S, S91.15-S91.159S, S91.25-S91.259S, S91.35-S91.359S, W53.01-W53.01XS, W53.11-W53.11XS, W53.21-W53.21XS, W53.81-W53.81XS, W54.0-W54.0XXS, W55.11-W55.11XS, W55.21-W55.21XS, W55.31-W55.31XS, W55.41-W55.41XS, W55.51-W55.51XS, W55.81W55.81XS, W56.01-W56.01XS, W56.11-W56.11XS, W56.21-W56.21XS, W56.31-W56.31XS, W56.41-W56.41XS, W56.51-W56.51XS, W56.81-W56.81XS, W58.01-W58.01XS, W58.11-W56.11XS, W59.01-W59.01XS, W59.11-W59.11XS, W59.21-W59.21XS, W59.81-W59.81XS, W61.01-W61.01XS, W61.11-W61.11XS,					

© 2024 Lehrer BJ et al. *JAMA Network Open*

Indication	ICD-10 codes	Standard Guidelines			Contemporary Guidelines[c]	
		Optimal Choice[a]	Optimal Duration[b] (days)	Reference	Optimal Duration[b] (days)	Reference
	W61.21-W61.21XS, W61.33-W61.33XS, W61.43-W61.43XS, W61.51-W61.51XS, W61.61-W65.61XS, W61.91-W61.91XS					
Appendicitis	K35 -K37	Amox-clav<br>Ceftriaxone<br>Ciprofloxacin<br>Levofloxacin<br>Metronidazole	7-14<br>7-14<br>7-14<br>7-14<br>7-14	Solomkin 2010 IDSA[17]	4-7<br>4-7<br>4-7<br>4-7<br>4-7	AAP 2021 Red Book[3]
Chronic sinusitis	J32-J32.9	Amoxicillin<br>Amox-clav<br>Clindamycin<br>Levofloxacin<br>Moxifloxacin<br>Metronidazole<br>TMP-SMX	21-42<br>21-42<br>21-42<br>21-42<br>21-42<br>21-42<br>21-42	Orlandi 2016[18]<br>Goldsmith 2003[19],<br>Brook 2017[20]	N/A	
Conjunctivitis	H10-H10.029, H10.2-H10.219, H10.23- 10.4-H10.409, H10.42-H10.429, H10.5-H10.509, H10.52-H10.529, H10.8, H10.89-H10.9, H16.2-H16.209, H16.29-H16.309	Amoxicillin<br>Amox-clav<br>Azithromycin	5<br>5<br>3-5	Azari 2013[21]	N/A	
Gastroenteritis	*(Cholera)*<br>A00-A00.9	Doxycycline	1	Connor 2023 CDC[22]	N/A	
	(Clostridium difficile)<br>A04.7, A04.71-A04.72	Fidaxomicin<br>Metronidazole<br>Vancomycin	10<br>10<br>10-14	McDonald 2018 IDSA[23]	N/A	
	(General)<br>A02.0, A02.25, A03-A05, A05.1-A05.3, A05.5-A05.9, A07.1-A08, A08.8-A09, R19.7	Azithromycin<br>Ceftriaxone<br>Ciprofloxacin<br>Vancomycin	1-3<br>1-3<br>1-3<br>10-14	Connor 2023 CDC[22],<br>Kim 2019[24]	N/A	
Genitourinary infection	*(Lower GU tract infection)*<br>N30-N30.21, N30.8-N30.91, N34-N34.2, N39, N45-N45.3, N47.6, N48.1-N48.22, N48.5, N76-	Amoxicillin<br>Amox-clav<br>Azithromycin<br>Cefixime	7-10<br>7-10<br>1<br>7-10	Roberts 2011[1],<br>Gupta 2011[2],<br>Workowsk 2021 CDC[11]	N/A	

© 2024 Lehrer BJ et al. *JAMA Network Open*

Indication	ICD-10 codes	Standard Guidelines			Contemporary Guidelines[c]	
		Optimal Choice[a]	Optimal Duration[b] (days)	Reference	Optimal Duration[b] (days)	Reference
	N76.3, N76.5-N75.6, N76.89, N77-N77.1, R30.0, R36-R36.0, R36.9	Cefpodoxime	7-10			
		Ceftriaxone	7-10			
		Cephalexin	7-10			
		Doxycycline	1-7			
		Metronidazole	1-7			
		Nitrofurantoin	7-10			
		Penicillin G	1-3			
		TMP-SMX	7-10			
	(Upper GU tract infection) N70-N70.03, N70.13-N74	Cefoxitin	1	Workowski 2021 CDC[11]		
		Ceftriaxone	1			
		Doxycycline	14			
		Metronidazole	14			
Lymphadenitis	I88.0-I88.9, L04.0-L04.9	Amoxicillin	10	Dulin 2008 AAFP[25]	5-7	AAP 2021 Red Book[3]
		Amox-clav	10		5-7	
		Cephalexin	10		5-7	
		Clindamycin	10		5-7	
Otitis externa	H60, H60.2-H60.399, H60.8-H60.93, H62--H62.43	Cephalexin	10	Sander 2001 AAFP[26]	N/A	
		Clindamycin	10			
		Ciprofloxacin	10			
		Levofloxacin	10			
		Moxifloxacin	10			
		Cefuroxime	10			
Otitis media	*(General otitis media and myringitis)* A380, H66-H66.003, H66.009-H66.013, H66.019, H66.4-H66-H67.9, H73-H73.099, H73.2-H73.23	Amoxicillin	5-10	Lieberthal 2013 AAP[27]	Based on age[d]	AAP 2021 Red Book[3]
		Amox-clav	5-10			
	(Recurrent episode) H66.04-H66.07, H66.014-H66.017	Amoxicillin	5-10	Lieberthal 2013 AAP[27] Pediatric Outpatient Treatment Table 2023 CDC[12]	Based on age[d]	AAP 2021 Red Book[3]
		Amox-clav	5-10			
		Ceftriaxone	1-3			
		Clindamycin	5-10			
Pharyngitis	A38-A38.0, J02-J03.91	Amoxicillin	10	Shulman 2012 IDSA[28]	N/A	
		Penicillin G	10			

© 2024 Lehrer BJ et al. *JAMA Network Open*

Indication	ICD-10 codes	Standard Guidelines			Contemporary Guidelines[c]	
		Optimal Choice[a]	Optimal Duration[b] (days)	Reference	Optimal Duration[b] (days)	Reference
		Penicillin V	10	Pediatric Outpatient Treatment Table 2023 CDC[12]		
Acute Sinusitis	*(Acute episodes)* J01-J01.00, J01.1-J01.10, J01.2-J01.20, J01.3-J01.30, J01.4-J01.40, J01.8-J01.80, J01.9-J01.90	Amoxicillin<br>Amox-clav	10-14<br>10-14	Chow 2012 IDSA[29]<br>Pediatric Outpatient Treatment Table 2023 CDC[12]	5-7<br>5-7	AAP 2021 Red Book[3]
	(Recurrent episodes) J01.01, J01.11, J01.21, J01.31, J01.41, J01.81, J01.91	Amoxicillin<br>Amox-clav<br>Clindamycin<br>Doxycycline<br>Levofloxacin	10-14<br>10-14<br>10-14<br>10-14<br>10-14	Chow 2012 IDSA[29]	N/A	
Skin and soft tissue infections	*(Cutaneous Abscess, Lymphangitis)* H20.05-H20.059, H60.0-H60.03, I89.1, J34.0, L02-L02.93, L03-L03.0, L03.02-L03.029, L03.04-L03.1,L03.12-L03.21, L03.22, L03.222-L03.3, L03.32-L03.8, L03.89-L03.9, L03.91, N61.1, N75.1, N76.4	Amoxicillin<br>Amox-clav<br>Cephalexin<br>Clindamycin<br>Dicloxacillin<br>Doxycycline<br>TMP-SMX	5-10<br>5-10<br>5-10<br>5-10<br>5-10<br>5-10<br>5-10	Stevens 2014 IDSA[16]	5-7<br>5-7<br>5-7<br>5-7<br>5-7<br>5-7<br>5-7	AAP 2021 Red Book[3]
	(Deep tissue abscesses) M65.0-M65.08, M65.1-M65.19, M71.0-M71.19	Amoxicillin<br>Amox-clav<br>Cephalexin<br>Clindamycin<br>Dicloxacillin<br>Doxycycline<br>TMP-SMX	7-14<br>7-14<br>7-14<br>7-14<br>7-14<br>7-14<br>7-14	Stevens 2014 IDSA[16]<br>Sexton DJ 2022[30]<br>Khodaee 2017[31]	N/A	
	(Cellulitis; Cellulitis-like) A46, H60.1-H60.13, H61.0-H61.039, L03.01-L03.019, L03.03-L03.039, L03.11-L03.119, L03.211-L03.213, L03.221, L03.31-L03.319,	Amoxicillin<br>Amox-clav<br>Cephalexin<br>Clindamycin	5<br>5<br>5<br>5	Stevens 2014 IDSA[16]	N/A	

© 2024 Lehrer BJ et al. *JAMA Network Open*

Indication	ICD-10 codes	Standard Guidelines Optimal Choice[a]	Optimal Duration[b] (days)	Reference	Contemporary Guidelines[c] Optimal Duration[b] (days)	Reference
	L03.81-L03.818, L03.90, L08.89-L08.9, L60.0, L73, L73.8-L73.9, W50	Dicloxacillin<br>Doxycycline<br>TMP-SMX	5<br>5<br>5			
	(Staphylococcal Scalded Skin Syndrome)<br>L00	Amoxicillin<br>Amox-clav<br>Cephalexin<br>Clindamycin<br>Dicloxacillin<br>Doxycycline<br>TMP-SMX	7-14<br>7-14<br>7-14<br>7-14<br>7-14<br>7-14<br>7-14	Stevens 2014 IDSA[16]<br>Braunstein 2014[32]	N/A	
	(Hidradenitis suppurativa)<br>L73.2	Clindamycin<br>Doxycycline	14<br>14	Stevens 2014 IDSA[16]<br>Ingram 2022[33]	N/A	
	(Impetigo, Hordeolum)<br>H00-H01, L01-L01.1	Amoxicillin<br>Amox-clav<br>Cephalexin<br>Clindamycin<br>Dicloxacillin<br>Doxycycline<br>TMP-SMX	5-7<br>5-7<br>5-7<br>5-7<br>5-7<br>5-7<br>5-7	Stevens 2014 IDSA[16]	N/A	
Excluded Tier 2 Diagnoses	A02.20, A02.8-A02.9, A06.0-A07.0, A38.1-A38.9, B47, B47.9, B60.2, B90-B90.9, B92-B94.0, B94, B94.8-B94.9, B96.21-B96.23, B99-B99.9, D59.3-D59.39, D70.3-D70.9, D73.0-D73.1, D73.3-D73.4, D80.2-D80.5, D80.7-D81.2, D81.89-D83.9, D84.821, G04.39-G04.81, G04.89-G05.4, H01.0-H01.02B, H04-H04.029, H04.3-H04.429, H05-H05.0, H10.43-10.439, H16-H16.119, H16.14-H16.149, H16.31-H16.329, H16.39-H16.399, H16.8-H16.9, H20.03-H20.039, H21.33-H21.339, H33.12-H33.129, H44.12-H44.129, H59.4-H59.43, H60.6-H60.63, H66.1-H66.3X9, H73.1-H73.13, H75.8-H75.83, H94.0-H94.03, I30.1, I31.31, I33-I33.9, I40-I41, I51.4, J10.0-J10.00, J11.0-J11.00, J44-J44.1, J95.02, J98.5, K05.3-K05.329, K11.22-K11.23, K12, K12.3-K12.30, K27, K67-K68, K85, K85.02, K85.12, K85.22, K85.32, K85.82, K85.92, K90.1, K91.85-K91.858, L05-08.82, L30.3, L66.3, L88, M46, M60.8-M60.9, M13.7-N13.9, N61.0, N99.521, N99.531, all infections from O00-09A.53, P00.82, P02.7, P02.78, R10.0, R10.82, R18, S05.2-S05.62XS					
**Tier 3 Diagnoses – Antibiotics are nearly never indicated**						
	All ICD-10 codes not specified above					

[a] Amox-clav = amoxicillin-clavulanate; TMP-SMX = Trimethoprim- sulfamethoxazole

[b] All durations less than or equal to the maximum listed duration were considered optimal for our study

© 2024 Lehrer BJ et al. *JAMA Network Open*

[c] N/A = not applicable

[d] Contemporary durations for acute otitis media were stratified by age (<2 years old = maximum duration of 10 days; ≥2 years old = maximum duration 7 days)

© 2024 Lehrer BJ et al. *JAMA Network Open*

eFigure 1. Hierarchy of ICD-10 Codes When Assigning Single Visit Diagnosis to Each Encounter

Prioritized Less

Pregnancy-related: O98812, O8612, O8621

Tier 3 Diagnoses: U070, R151, J180, H0011, H0012, H0013, H0014, H0015, H0016, J129, J1289, J208, A0839, A084, A082, A0811, A080, A069, R197

Broad Tier 1 or 2 Diagnoses: B9689, A498, A499, Z20818, B9620, N762, N711, N760, R300, L081, A6920, K1120, K120, K121, K122, H01001, H01002, H01003, H01004, H01005, H01006, H0101A, H0100A, H0100B, H0102A, J690, J440, J441, A419, R6521, A280, A3700, A029, A491, A408, J13, B953, B950, B963, B961, B965, J9502, A4151, P362, B958, B957, B9561, A4901, A4902, B9562, B952, A493

Tier 2 Diagnoses Primarily: L738, L739, L0889, L089, L709, L708, L700, A071, A041, A072, A040, A039, B9621, A059, A038, A09, A058, A045, A020, A0471, A0472, B9681, T8141XA, T84623A, T8469XA, T8142XD, T8149XA, H109, H10022, H10023, H1030, H1031, H1032, H1033, H00019, H00029, H00024, H00026, H00014, H00015, H00016, H00012, H00011, H00039, H00034, H00032, H00033, H00036, H16141, L0293, L0233, L0212, L02224, J340, L0202, L0223, L02222, L02223, L0232, L0292, L02421, L02422, L02221, L02424, L02425, L02429, L03315, L0390, L03011, L03319, L03012, L03019, N4822, L03031, L03032, L03039, L02811, L02512, L02213, L03112, L03113, L03114, L03115, L03116, L03116, L03119, L03211, L03221, L03811, L03818, L03311, H61001, H6011, H6012, L03314, L03317, L03316, A389, L0100, L0101, L0101, L0103, L0109, A483, J028, J029, J020, J0300, J0390, J0391, J0380, J0301, N610, N764, L0591, L0501, L0502, L0231, H6001, H6002, L02818, L02419, M65052, L02219, L0291, L02415, L0201, L0211, L02212, L02211, L02214, L02411, L02412, L02413, L02414, L02415, L02416, L02511, L02611, L02612, N611, L02216, L080, L732, H60393, S31159A, S0087XA, S61551A, S61251A, S61259A, S61252A, S01459A, S0195XA, S01451A, S01551A, S0185XA, S81851A, S81852A, S80872A, S71152A, S51851A, S51852A, S61250A, S81052A, S41151A, S41152A, S61451A, S71152A, S71151A, S0105XA, S0125XA, S3125XA, S01152A, S61256A, S61051A, S60571A, W5531XA, W5581XA, W5541XA, W5311XA, W5381XA, S91351A, S91052A, S61552A, S61452A, W5501XA, W503XXA, W540XXA, W5401XA, H73019, H73011, H73012, H73002, H608X3, H60312, H608X2, H6240, H6241, H6242, H60333, H60331, H60332, H60391, H60319, H60392, H6090, H6091, H6092, H6093, H60313, H671, H672, H673, H66001, H66002, H66003, H66009, H66011, H66012, H66013, H6641, H6642, H6643, H6690, H6691, H6692, H6693, H66004, H66005, H66006, H66007, H66014, H66016, H6613, H663X1, H663X2, H663X3, L042, L048, L03041, L03042, I891, L0391, L041, L043, I889, L049, L040

Tier 1 Diagnoses Primarily: R7881, J156, A3711, J15211, J15212, J14, J189, J159, J158, J188, J168, J181, J160, J157, J869, J860, J851, J84116, D5701, D57211, J0180, J0190, J0100, J0110, J0120, J0140, J0141, J0181, J0111, J0191, J0101, J321, J322, J320, J324, J329, N3001, N3000, N3080, N3090, N3091, N3010, N3020, N3021, O2301, O2340, O2341, O2342, O2342, O2343, O2303, O23591, O23592, O23593, O23599, N390, N341, N342, R369, N451, N7093, N7001, N452, N766, N761, Z202, Z113, A64, A5489, O98211, O98212, O98213, A549, A5400, A5401, A5402, A5403, A5409, A749, A7481, A5600, A5601, A5602, A5609, A5619, A562, A568, A5900, A5901, A599, A5149, A5424, A5611, N733, N739, N72, L03213, H05019, H05011, H05012, P393, L00, K1230, K122, J391, K047, J36, J390, K91850, K612, K5700, N151, N136, N111, N110, N10, K5530, I96, K650, K8592, K37, K3580, K35890, K35891, K3530, K3520, K3521, K3532, K3533, K631, K651, K658, K611, K610, K750, P381, P381, K50914, K50114, J9851, M609, M6008, M60061, T80212A, T80211A, T8571XA, I00, M00869, M0230, M02361, M02362, M00861, M00871, M009, M00122, M00252, M00022, M00062, H70001, H70002, H70003, H7091, H7092, H7093, H7013, H7012, M8620, M868X7, M86122, M86172, M868X8, M86171, M86622, M8618, M868X2, M868X6, M86131, M86139, M869, M86121, M869, M4624, M4628, M4620, M868X5, M86151, M86152, M86161, M86162, M8608, M86111, M8618, M86612, M4624, M868X6, M869, M86662, G0490, G039, G001, G002, G008, G009, I38, I330, G062, G060, A184, A157

Prioritized More

Hierarchy reads left to right and top to bottom.

List of ICD-10 codes is not comprehensive; this figure only shows ICD-10 codes that were assigned by at least one provider in our dataset. To see list of excluded visit diagnoses, see eTable 1.

© 2024 Lehrer BJ et al. *JAMA Network Open*

eFigure 2. Algorithm for Determining Optimal Antibiotics for Each Encounter

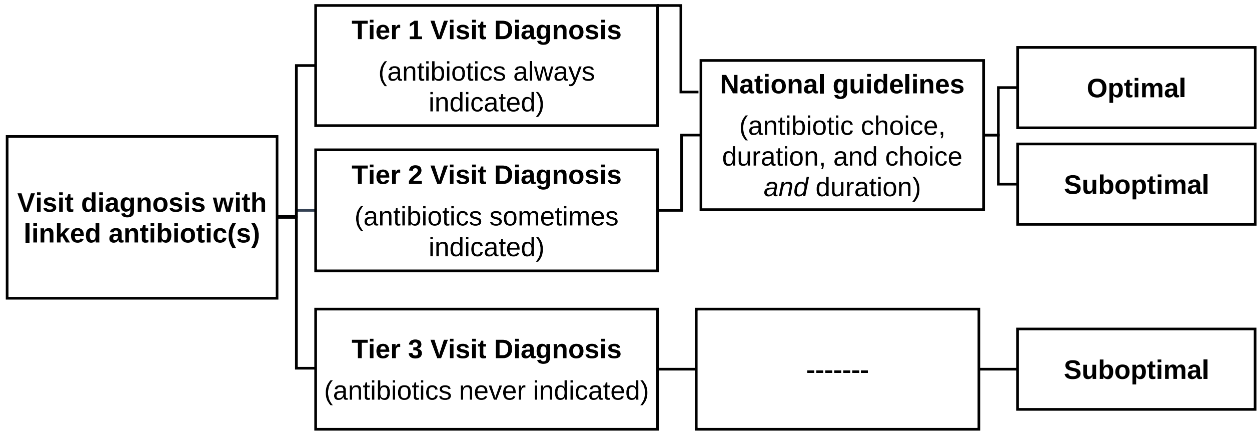

© 2024 Lehrer BJ et al. *JAMA Network Open*

eFigure 3. Most Common Diagnoses and the Suboptimal Antibiotics That Were Prescribed Instead of Optimal Therapies (Based on Antibiotic Choice)

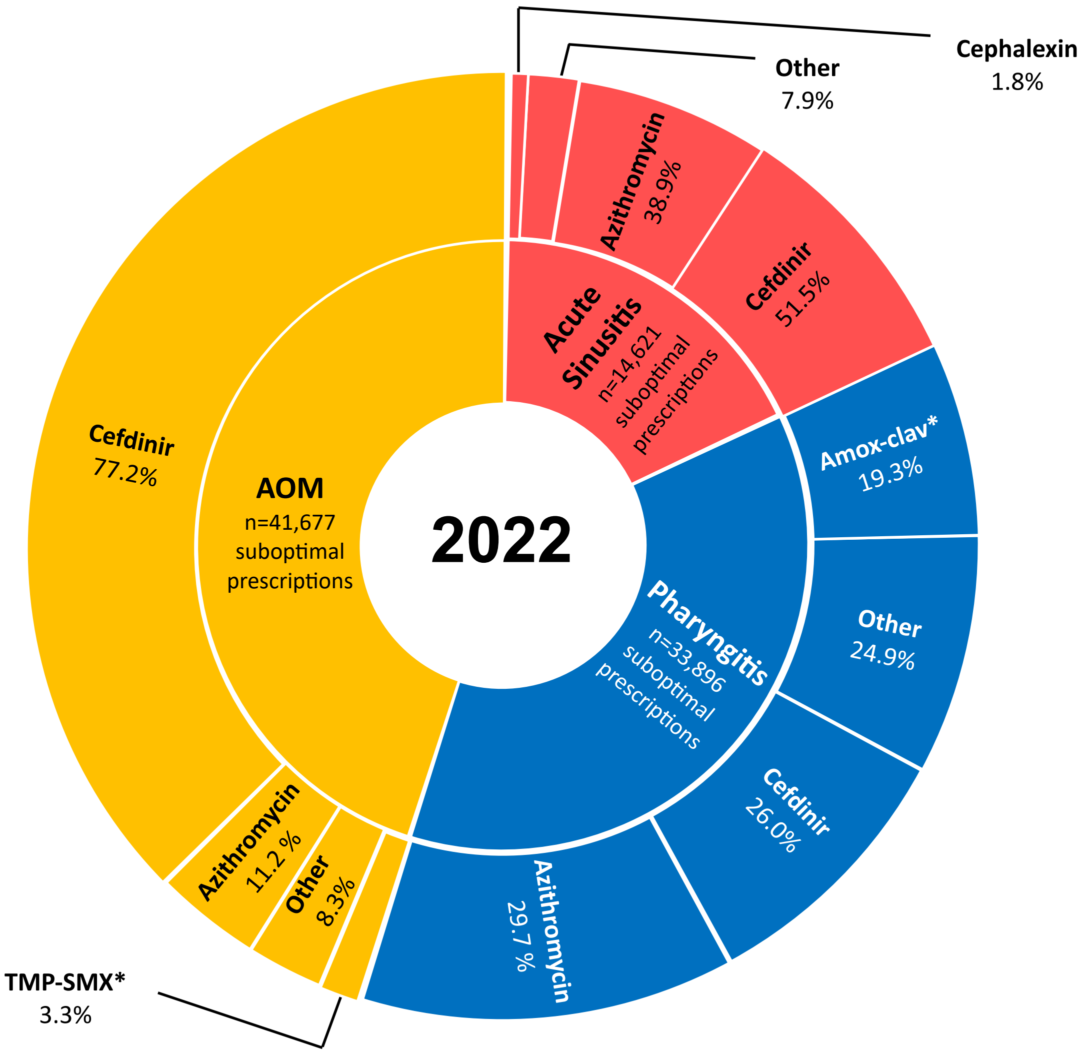

*TMP-SMX = trimethoprim-sulfamethoxazole; Amox-clav = amoxicillin-clavulanate

© 2024 Lehrer BJ et al. *JAMA Network Open*

eTable 2. Most Common Tier 3 Diagnoses With At Least 1 Antibiotic Prescribed

ICD-10 Code	Description of ICD-10 Code	# of Encounters with at least 1 Antibiotic Prescribed[1]
J06.9	Acute upper respiratory infection, unspecified	20,388
R50.9	Fever, unspecified	19,114
Z20.822	Contact with and (suspected) exposure to COVID-19	15,446
R05.9	Cough, unspecified	12,332
Z00.129	Encntr for routine child health exam w/o abn findings	7,865
Z23	Encounter for immunization	6,766
R05.1	Acute cough	5,682
F84.0	Autistic disorder	4,688
R09.81	Nasal congestion	4,409
B34.9	Viral infection, unspecified	4,278
Z68.52	Body mass index pediatric, 5th percentile to <85% for age	3,766
J30.1	Allergic rhinitis due to pollen	3,718
J20.9	Acute bronchitis, unspecified	3,693
J30.89	Other allergic rhinitis	3,389
F90.2	Attention-deficit hyperactivity disorder, combined type	3,321
Z71.3	Dietary counseling and surveillance	3,145
U07.1	COVID-19	3,073
Z20.828	Contact w/ and expos to oth viral communicable diseases	3,051

Abbreviations: abn = abnormal; encntr = encounter; expos = exposure; oth = other; w/ = with; w/o = without

[1]Table only includes diagnoses with >3000 encounters

© 2024 Lehrer BJ et al. *JAMA Network Open*

eReferences

1. Roberts KB, Subcommittee on Urinary Tract Infection SC on QI and M. Urinary Tract Infection: Clinical Practice Guideline for the Diagnosis and Management of the Initial UTI in Febrile Infants and Children 2 to 24 Months. *Pediatrics*. 2011;128(3):595-610. doi:10.1542/peds.2011-1330

2. Gupta K, Hooton TM, Naber KG, et al. International Clinical Practice Guidelines for the Treatment of Acute Uncomplicated Cystitis and Pyelonephritis in Women: A 2010 Update by the Infectious Diseases Society of America and the European Society for Microbiology and Infectious Diseases. *Clin Infect Dis*. 2011;52(5):e103-e120. doi:10.1093/cid/ciq257

3. Committee on Infectious Diseases AA of P, Kimberlin DW, Barnett ED, Lynfield R, Sawyer MH, eds. Systems-based Treatment Table. In: *Red Book: 2021–2024 Report of the Committee on Infectious Diseases*. American Academy of Pediatrics; 2021:0. doi:10.1542/9781610025782-S4_012

4. Jones NL, Koletzko S, Goodman K, et al. Joint ESPGHAN/NASPGHAN Guidelines for the Management of Helicobacter pylori in Children and Adolescents (Update 2016). *J Pediatr Gastroenterol Nutr*. 2017;64(6):991-1003. doi:10.1097/MPG.0000000000001594

5. Nguyen DH, Martin JT. Common Dental Infections in the Primary Care Setting. *Am Fam Physician*. 2008;77(6):797-802.

6. Cionca N, Giannopoulou C, Ugolotti G, Mombelli A. Amoxicillin and Metronidazole as an Adjunct to Full-Mouth Scaling and Root Planing of Chronic Periodontitis. *J Periodontol*. 2009;80(3):364-371. doi:10.1902/jop.2009.080540

7. Bradley JS, Byington CL, Shah SS, et al. The Management of Community-Acquired Pneumonia in Infants and Children Older Than 3 Months of Age: Clinical Practice Guidelines by the Pediatric Infectious Diseases Society and the Infectious Diseases Society of America. *Clin Infect Dis Off Publ Infect Dis Soc Am*. 2011;53(7):e25-e76. doi:10.1093/cid/cir531

8. Bielicki JA, Stöhr W, Barratt S, et al. Effect of Amoxicillin Dose and Treatment Duration on the Need for Antibiotic Re-treatment in Children With Community-Acquired Pneumonia: The CAP-IT Randomized Clinical Trial. *JAMA*. 2021;326(17):1713-1724. doi:10.1001/jama.2021.17843

9. Pernica JM, Harman S, Kam AJ, et al. Short-Course Antimicrobial Therapy for Pediatric Community-Acquired Pneumonia: The SAFER Randomized Clinical Trial. *JAMA Pediatr*. 2021;175(5):475-482. doi:10.1001/jamapediatrics.2020.6735

10. Williams DJ, Creech CB, Walter EB, et al. Short- vs Standard-Course Outpatient Antibiotic Therapy for Community-Acquired Pneumonia in Children: The SCOUT-CAP Randomized Clinical Trial. *JAMA Pediatr*. Published online January 18, 2022. doi:10.1001/jamapediatrics.2021.5547

11. Workowski KA, Bachmann LH, Chan PA, et al. Sexually Transmitted Infections Treatment Guidelines, 2021. 2021;70(4).

12. Pediatric Outpatient Treatment Recommendations | Antibiotic Use | CDC. Published June 15, 2023. Accessed March 4, 2024. https://www.cdc.gov/antibiotic-use/clinicians/pediatric-treatment-rec.html

13. Zaoutis T, Shaikh N, Fisher BT, et al. Short-Course Therapy for Urinary Tract Infections in Children: The SCOUT Randomized Clinical Trial. *JAMA Pediatr*. 2023;177(8):782-789. doi:10.1001/jamapediatrics.2023.1979

14. Johnson BA, Nunley JR. Use of Systemic Agents in the Treatment of Acne Vulgaris. *Am Fam Physician*. 2000;62(8):1823-1830.

15. Zaenglein AL, Pathy AL, Schlosser BJ, et al. Guidelines of care for the management of acne vulgaris. *J Am Acad Dermatol*. 2016;74(5):945-973.e33. doi:10.1016/j.jaad.2015.12.037

16. Stevens DL, Bisno AL, Chambers HF, et al. Practice Guidelines for the Diagnosis and Management of Skin and Soft Tissue Infections: 2014 Update by the Infectious Diseases Society of America. *Clin Infect Dis*. 2014;59(2):e10-e52. doi:10.1093/cid/ciu296

17. Solomkin JS, Mazuski JE, Bradley JS, et al. Diagnosis and Management of Complicated Intra-abdominal Infection in Adults and Children: Guidelines by the Surgical Infection Society and the Infectious Diseases Society of America. *Clin Infect Dis*. 2010;50(2):133-164. doi:10.1086/649554

18. Orlandi RR, Kingdom TT, Hwang PH, et al. International Consensus Statement on Allergy and Rhinology: Rhinosinusitis. *Int Forum Allergy Rhinol*. 2016;6(S1):S22-S209. doi:10.1002/alr.21695

19. Goldsmith AJ, Rosenfeld RM. Treatment of pediatric sinusitis. *Pediatr Clin North Am*. 2003;50(2):413-426. doi:10.1016/S0031-3955(03)00027-0

20. Brook I. The role of antibiotics in pediatric chronic rhinosinusitis. *Laryngoscope Investig Otolaryngol*. 2017;2(3):104-108. doi:10.1002/lio2.67

21. Azari AA, Barney NP. Conjunctivitis. *JAMA J Am Med Assoc*. 2013;310(16):1721-1729. doi:10.1001/jama.2013.280318

22. Travelers' Diarrhea | CDC Yellow Book 2024. Accessed December 17, 2023. https://wwwnc.cdc.gov/travel/yellowbook/2024/preparing/travelers-diarrhea

23. McDonald LC, Gerding DN, Johnson S, et al. Clinical Practice Guidelines for Clostridium difficile Infection in Adults and Children: 2017 Update by the Infectious Diseases Society of America (IDSA) and Society for Healthcare Epidemiology of America (SHEA). *Clin Infect Dis*. 2018;66(7):e1-e48. doi:10.1093/cid/cix1085

24. Kim YJ, Park KH, Park DA, et al. Guideline for the Antibiotic Use in Acute Gastroenteritis. *Infect Chemother*. 2019;51(2):217-243. doi:10.3947/ic.2019.51.2.217

25. Dulin MF, Kennard TP, Leach L, Williams R. Management of Cervical Lymphadenitis in Children. *Am Fam Physician*. 2008;78(9):1097-1098.

© 2024 Lehrer BJ et al. *JAMA Network Open*

26. Sander R. Otitis Externa: A Practical Guide to Treatment and Prevention. *Am Fam Physician*. 2001;63(5):927-937.

27. Lieberthal AS, Carroll AE, Chonmaitree T, et al. The Diagnosis and Management of Acute Otitis Media. *Pediatrics*. 2013;131(3):e964-e999. doi:10.1542/peds.2012-3488

28. Shulman ST, Bisno AL, Clegg HW, et al. Executive Summary: Clinical Practice Guideline for the Diagnosis and Management of Group A Streptococcal Pharyngitis: 2012 Update by the Infectious Diseases Society of America. *Clin Infect Dis*. 2012;55(10):1279-1282. doi:10.1093/cid/cis847

29. Chow AW, Benninger MS, Brook I, et al. IDSA Clinical Practice Guideline for Acute Bacterial Rhinosinusitis in Children and Adults. *Clin Infect Dis*. 2012;54(8):e72-e112. doi:10.1093/cid/cis370

30. Sexton DJ, Leversedge FJ. Infectious tenosynovitis. In: *UpToDate*. Wolters Kluwer; 2022.

31. Khodaee M. Common Superficial Bursitis. *Am Fam Physician*. 2017;95(4):224-231.

32. Antibiotic Sensitivity and Resistance Patterns in Pediatric Staphylococcal Scalded Skin Syndrome - PMC. Accessed March 9, 2024. https://www.ncbi.nlm.nih.gov/pmc/articles/PMC4349361/

33. Ingram J. Hidradenitis suppurativa: Management. In: *UpToDate*. Wolters Kluwer; 2022.

© 2024 Lehrer BJ et al. *JAMA Network Open*
