# Supplementary material for: Optimal Pediatric Outpatient Antibiotic Prescribing
Source: JAMA Netw Open. 2024 Oct 3;7(10):e2437409. doi: 10.1001/jamanetworkopen.2024.37409 (PMC11450517; doi:10.1001/jamanetworkopen.2024.37409)
Supplement: Supplement 2. — Data Sharing Statement [file jamanetwopen-e2437409-s002.pdf]

Data Sharing Statement

Lehrer. Optimal Pediatric Outpatient Antibiotic Prescribing. *JAMA Netw Open*. Published October 03, 2024. doi:10.1001/jamanetworkopen.2024.37409

Data
Data available: Yes
Data types: Deidentified participant data, Data dictionary
How to access data: sophie.e.katz@vumc.org
When available: With publication

Supporting Documents
Document types: None

Additional Information
Who can access the data: researchers whose proposed use of the data has been approved
Types of analyses: For those conducting research in the antimicrobial stewardship space
Mechanisms of data availability: with a signed data access agreement
Any additional restrictions: None
